# Mature Ovarian Teratoma: Neurological Implications in a Young Woman

**DOI:** 10.1155/2021/3085559

**Published:** 2021-11-05

**Authors:** Michele Carlo Schiavi, Fabio Manganelli, Claudia Morgani, Pietro Cignini, Veronica Yacoub, Valerio Carletti, Debora Grilli, Anna Di Pinto, Francesco Bisogni, Azzurra Ligato, Francesco Galanti, Herbert Carmelo Carlo Valensise, Pier Luigi Palazzetti

**Affiliations:** ^1^Department of Obstetrics and Gynecology, “Sandro Pertini” Hospital, Rome, Italy; ^2^Department of Obstetrics and Gynecology, “Tor Vergata” University, Rome, Italy; ^3^Department of Obstetrics and Gynecology, “Santa Maria Goretti” Hospital, Latina, Italy; ^4^Department of Obstetrics and Gynecology, “Casilino” Hospital, Rome, Italy

## Abstract

Anti-NMDAR encephalitis is an autoimmune syndrome associated with antibodies against NMDA receptors. In some cases, it is associated with various tumors; one of them is ovarian teratoma, which mostly affects women below the age of 30 years. Here, we report a case of ovarian teratoma associated with anti-NMDAR encephalitis treated with both laparoscopic surgery and immunotherapy. Multidisciplinary approach is the cornerstone for the management of this syndrome.

## 1. Introduction

Anti-N-methyl-D-aspartate receptor (NMDAR) encephalitis is a rare paraneoplastic syndrome associated with various tumors, most commonly with ovarian teratoma. It is associated with antibodies against NR1–NR2 heteromers of the NMDA receptor which can be detected in cerebrospinal fluid (CSF) and serum [1]. Patients with anti-NMDAR encephalitis manifest psychiatric symptoms, usually preceded by fever and headache, including restlessness and severe central hypoventilation which often requires ventilator support. It also can cause oral facial dyskinesia, affective disturbance, psychosis, hallucination, memory loss, seizures, vegetative deregulation, and autonomic dysfunction [2]. In several cases, the electroencephalogram of these patients reveals diffuse delta slowing waves. MRI examination may produce normal results or abnormally high signals in the cerebral cortex, cerebellum, or medial temporal lobe. This disease is closely associated with the development of ovarian teratoma, so histological and antibody examination is also needed to confirm the diagnosis [3]. Basic therapeutic management for anti-NMDAR encephalitis mainly includes tumor resection and immune therapy. First-line medical therapy includes steroid, intravenous immunoglobulin (IVIG), plasmapheresis, and monoclonal antibodies, such as rituximab. Previous studies showed a significantly better outcome in patients with early resection of teratomas, and the same result included those patients treated with early use of corticosteroids and IgG-depleting strategies (IVIG or plasma exchange) [4]. The real incidence of this entity is unknown, but it was diagnosed in almost 0.85% of the women operated on for ovarian teratoma [5]. Mature cystic teratomas (or dermoid cysts) are ovarian neoplasm which contains mature tissue components originating from two or three germinal layers. These tumors are more often cystic and can reach large diameters. It can lead to anti-N-methyl-D-aspartate receptor (NMDAR) encephalitis, but its exact role in the pathogenesis is not clear yet. It is hypothesized that the glial cells present within the teratoma produce antibodies to NMDAR, which in turn cause severe encephalitis. The cause of that antibody production is still unknown. The targets are NR1 and NR2 subunits at NMDA receptors, which cause reduced synaptic plasticity. This change reduces NMDA receptor activity, which affects cognitive and behavioural deficits leading to schizophrenia and psychosis [3]. Histologic markers of atypical glioneuronal cells (resembling cells from gangliogliomas or ganglioneuroblastomas) were found in teratoma tissue from anti-NMDAR encephalitis patients but not from controls: it suggests that specific neural antigens present in ovarian teratomas cause a pathogenic immune response [6]. Anyway, it is clear that the removal of this teratoma will stop the production of the antibody [3]. Here, we report a case of a young patient who presented anti-NMDAR encephalitis associated with ovarian teratoma. The case was treated with a multidisciplinary approach, leading to a complete resolution of all symptoms.

## 2. Case Report

A 25-year-old nulliparous Italian female patient was admitted to the Intensive Care Unit of Sandro Pertini Hospital, Rome, Italy, on April 4, 2020. The patient started to have body temperature of 38.1°C, headache, decreased consciousness, repetitive talking, and involuntary movements on mouth and feet reported from March 28, 2020. After 3 days, she developed amnesia, followed by delirium and discontinuous confusion. On April 4, 2020, the patient had started an epileptic seizure that lasted for few minutes. After this stage, the patient was admitted to the emergency department of Sandro Pertini Hospital of Rome for the appropriate investigations and therapy. Physical examination upon admission revealed a decreased Glasgow Coma Scale (eye response: 3; verbal response: 3; and movement response: 4), blood pressure was 139/68 mmHg, pulse was 90 times per minute, respiratory rate was 16 times per minute, and body temperature was 38.3°C. Neurological examination revealed severe nuchal rigidity, spastic tetraparesis, brisk reflexes, increased muscle tone of upper and lower limbs, Babinski reflex bilaterally positive, and numerous comitial seizures. Moreover, during hospital course, the patient developed numerous complex partial seizures. Pleocytosis was detected in the cerebrospinal fluid (CSF). Electroencephalography revealed frequency 7-9 c/s unstable, irregular, hyporeagent, and symmetric intermixed with delta activity at 1-2 c/s especially anterior regions and periventricular. Diagnostic suspicion included viral encephalitis, autoimmune encephalitis, and meningoencephalitis. Brain magnetic resonance imaging (MRI) scans showed periventricular, subarachnoid, and cortical hyperintensity on fluid-attenuated inversion recovery (FLAIR). Collaterally, a total body CT scan revealed a 22 × 8 × 19 mm dermoid cyst of the right ovary. Following radiological investigation, the diagnostic suspect of autoimmune encephalitis led to send patient's CSF and serum's sample for the NMDAR antibody test in the oncoimmunology center. Meanwhile, abdominal ultrasound (US) was performed to find link between dermoid cyst and neurological symptoms. Transvaginal ultrasound revealed a right adnexal diffusely echogenic unilocular mass sized 23 × 18 × 11 mm with posterior sound attenuation due to sebaceous material and hair within the cyst cavity, mural hyperechoic Rokitansky nodule, echogenic shadowing dental components, no internal vascularization at the color-Doppler examination. The lesion was considered to be teratoma. To relive worsening neurological symptoms, she was initially treated with anticonvulsants (oxcarbazepine, phenytoin, phenobarbital, and levetiracetam), neuroprotector, intravenous methylprednisolone, 6 courses of plasmapheresis, and 5 courses of IVIG.

CSF NMDAR antibody returned positive confirming diagnosis of paraneoplastic NMDAR antibody-associated limbic encephalitis. With multidisciplinary team meeting (Neurologist, Gynecologist, Internist, Intensivist, and Anesthesiologist), confirming diagnostic suspicion, it was decided to undertake further surgical treatment and remove the ovarian lesion. Laparoscopic resection of the right ovary was performed on May 5, 2020. A postoperative pathologic examination revealed the following: right ovarian mass of primitive neuroglial elements mingled within surrounding lymphocytes and ectodermal elements, including squamous epithelium, sebaceous tissue, and hair elements. Therefore, pathological examination confirmed the diagnosis of mature teratoma. The patient's conditions gradually improved after the surgical treatment. The absence of remaining teratoma tissue was confirmed in a follow-up abdominopelvic CT. After the surgery, first-line medical therapy including steroid, intravenous immunoglobulin (IVIG) for 5 days, phenobarbital, valproate, levetiracetam, and olanzapine to control the epileptic and mental symptoms was performed. Following combined medical and surgical therapy, the patient showed significant improvement in language and cognitive function. The complete recovery was achieved after psychomotor rehabilitation sessions, including physiokinesitherapy and hydrotherapy, and behavioural rehabilitation. One month later, the patient mobilized without involuntary movements or seizures. After three months, all mental symptoms improved and the patient gained perfect cognitive and language function. Therefore, the patient was discharged on June 4, 2020, and she was sent for 3 months to a rehabilitation facility for motor and psychiatric outcomes.

After this treatment, she discontinued all medications; she developed complete resolution of all cognitive and mood symptoms that persisted prior to surgery and did not experience any further symptoms.

## 3. Discussion

This clinical case showed a young patient affected by anti-NMDAR encephalitis, a paraneoplastic syndrome mostly related to the presence of ovarian teratoma, a unilocular cyst which contains cells originating from each germinal layer [3]. This syndrome is associated with the presence of antibodies against NR1 and NR2 heteromers of NMDA receptors produced by the glial cells found in teratoma tissue. These receptors play an important role in neuronal functions, such as synaptic plasticity, learning, and memory. Due to the presence of anti-NMDAR antibodies in blood and CSF, we observed in our patient severe nuchal rigidity, spastic tetraparesis, brisk reflexes, fever, and complex partial seizures, outlining a case of severe encephalitis. Anti-NMDAR encephalitis was first described in 2007 [7]. The real incidence of this paraneoplastic syndrome is still unknown.

Women's age seems to be a significant parameter: two recent reviews described an age range from 7 years old to 33 years old, with most cases occurring in very young women and in women below the age of 30 years [2, 8]. In 2011, a study of 501 patients with anti-NMDAR encephalitis found that 38% of the patients had concomitant tumors [9], and a recent review of 432 cases of anti-NMDAR encephalitis found that among 293 female patients, 23% was diagnosed with ovarian teratoma [5, 10]. The prognosis of anti-NMDAR encephalitis is usually better than other types of encephalitis: in any case, it depends on the early diagnosis and appropriate treatment. In literature are described dermoid cysts of different sizes, from 10 mm to very large 200 mm ones, and this information is clinically important because the management is often decided according to this parameter [1], although asymptomatic small dermoid cysts (typically less than 50 mm) are usually managed conservatively to maintain ovarian reserve; moreover, larger tumors are often resected to avoid complications such as adnexal torsion. It seems that anti-NMDAR encephalitis often occurs in the presence of very small cysts, often misrecognised, that would not have been otherwise resected [3]. For this reason, it is important to consider every small and asymptomatic dermoid cyst as a possible risk factor for paraneoplastic encephalitis. However, complete recovery of patients with this kind of encephalitis can be achieved only with surgical treatment: the removal of teratoma suppresses the production of antibodies with the resolution of symptoms [3]. For most young patients with mature teratoma, laparoscopic operation technique is highly recommended, considering the fertility requirements and protection of the ovary. Otherwise, in case of immature teratoma, salpingo-oophorectomy seems to be the best surgical approach to avoid residual disease [2, 8]. In literature, there is no comparative analysis between whole adnexectomy and exclusive lesion excision. Clinical analysis is insufficient to affirm which surgical therapy leads to the best prognosis, outcome, relapse-free period, and ovarian reserve. Therefore, there is still no standardization of both medical and surgical treatment. First-line immunotherapy uses steroids, which have the important role to prevent CNS damage, IVIG, and plasmapheresis. The rational use of IVIG is decreasing T cell proliferation and cytokine level and depressing B cell differentiation. Its effects are usually increased with the association of plasmapheresis, whom benefit is to remove antibodies and inflammation mediators. Rituximab function diminishes the CNS antibodies, including B cell, but it is used as a second line [3]. In our case, the patient achieved the complete resolution after laparoscopic resection and first-line immunotherapy.

In conclusion, autoimmune anti-NMDAR encephalitis associated with ovarian teratoma is a sporadic case that is challenging to manage. The clinical approach required experts in gynecology, neurology, physiatry, and intensive care to establish careful management. The positive response to both medical and surgical therapy underlines the importance of early diagnosis and multidisciplinary management. Further multicenter studies are needed to settle earlier and standardized diagnosis and treatment. Even if an increasing number of journals have reported on anti-NMDAR encephalitis in the recent years, in literature, there is no guideline for the postoperative rehabilitation and each patient has been treated with a different approach. Our case shows that multidisciplinary approach is the cornerstone for the management of this rare syndrome and new protocols for rehabilitation therapies are needed to achieve a complete recovery in a very short time and an improvement in neurological outcomes.
